# PAX2, PAX8, and PR are correlated with ovarian seromucinous borderline tumor with endometriosis

**DOI:** 10.1186/s13048-022-00975-5

**Published:** 2022-04-06

**Authors:** Bo Seong Yun, Seyeon Won, Ju-Hyun Kim, Nara Lee, Miseon Kim, Mi Kyoung Kim, Mi-La Kim, Yong Wook Jung, Ji Young Kim, Seok Ju Seong, Eunah Shin

**Affiliations:** 1 Department of Obstetrics and Gynecology, CHA Ilsan Medical Center, CHA University School of Medicine, Goyang, Korea; 2 Department of Obstetrics and Gynecology, CHA Gangnam Medical Center, CHA University School of Medicine, Seoul, Korea; 3 Department of Obstetrics and Gynecology, University of Ulsan College of Medicine, Asan Medical Center, Seoul, Korea; 4 Department of Pathology, CHA Gangnam Medical Center, CHA University School of Medicine, Seoul, Korea; 5 Department of Pathology, Yongin Severance Hospital, Yonsei University College of Medicine, 363, Dongbaekjukjeon-daero, Giheung-gu, Yongin-si, 16995 Gyeonggi-do Korea

**Keywords:** Borderline tumor, Seromucinous, Endometrioid, Endometriosis, PAX2, PAX8, PR

## Abstract

**Background:**

Ovarian “seromucinous carcinoma” has been recently removed in 2020 5^th^ Edition of WHO classification of Female Genital Tumors and is considered as a subtype of endometrioid carcinoma with mucinous differentiation, while “seromucinous borderline tumor” remains and exists as a distinct entity. Both diseases may be considered as no more same lineage. However, ovarian seromucinous borderline tumor (SMBT) is also one of the endometriosis-related neoplasm of ovary similar to endometrioid tumor, featuring that about 50% of ovarian SMBTs combine endometriosis. The present study was aimed to investigate whether the ovarian SMBTs are different in clinical features and molecular patterns, according to the presence of combined endometriosis.

**Results:**

There were no statistical differences in clinical findings between two groups. There was also no significant difference in pregnancy outcomes and recurrence between two groups. In immunohistochemical patterns, there was a statistically significant difference in PAX2 and PAX8 expression between in ovarian SMBT with or without endometriosis (*P* = 0.016, *P* < 0.001). Only a few cases of ovarian SMBT with endometriosis showed expression of PAX2 and conversely, most of the cases showed expression of PAX8. PR positivity was more prominent in ovarian SMBT with endometriosis than without endometriosis (*P* = 0.018), although there was no difference in positive ER expression. There were no statistical differences in WT1, CK20 and CDX2 expressions between two groups.

**Conclusions:**

Ovarian SMBT with endometriosis did not clinically differ from that without endometriosis. However, the molecular patterns were different between two groups and ovarian SMBT with endometriosis is close to endometrioid tumor types unlike SMBT without endometriosis. Further, a direct comparison study between seromucinous borderline tumor and endometrioid borderline tumor is needed with a gene profiling study to prove their relationship.

## Background

Ovarian seromucinous borderline tumor (SMBT) is the type that was newly adopted as a distinct diagnostic category of ovarian epithelial borderline tumors in 2014 WHO classification of ovarian tumors [1]. SMBT comprises two or more cell types of Müllerian origin, mostly endocervical-like mucinous cells combined with other various cell types, such as serous/ciliated, endometrioid, squamous, clear cell, hobnail, eosinophilic, or indeterminate, each in a different proportion [1]. In the past, it has been classified under the ovarian mucinous tumor as endocervical-like type [2]. However, it has papillary projections within the cyst, which is mostly unilocular, and microscopically it shows characteristic broad papillae lined by serous type epithelial cells with abundant eosinophilic cytoplasm admixed with a varying number of endocervical-like mucinous cells [3]. Moreover, it shows a very different clinical course from GI type mucinous borderline tumor, rendering reclassification of the tumor as an independent category from the mucinous tumor.

SMBT has some distinctively characteristic features. First, endometriosis is found in about 50% of ovarian SMBTs, unlike other types of borderline tumors. SMBT is considered as one of the endometriosis-related neoplasm of the ovary, although the underlying causal relationship between the two is not elucidated. Second, the seromucinous category of the ovarian epithelial tumors no longer has carcinoma counterpart in WHO classification. Recently, ovarian “seromucinous carcinoma” has been removed from the seromucinous category in 2020 5^th^ Edition of WHO classification of Female Genital Tumors, because its diagnostic reproducibility is poor and has a high proportion of molecular overlap with endometrioid carcinoma [4]. So instead, ovarian seromucinous carcinoma was removed from the seromucinous category and placed in endometrioid category as a subtype of endometrioid carcinoma with mucinous differentiation, while benign and borderline seromucinous tumors remains as a distinct entity.

Ovarian SMBTs consist of various cells of Mullerian origin and they also contain endometriosis in about 50%. However, little is known about the characteristics of ovarian SMBT and the reason for endometriosis-associated SMBT cases remains unknown. Therefore, we aimed to clarify whether the presence or absence of endometriosis in ovarian SMBTs show different characteristics, and thus validate the relationship between SMBT and endometriosis. There is scarce evidence exploring whether SMBTs differ clinically and molecularly according to the presence or absence of combined endometriosis.

We aimed to investigate whether the ovarian SMBTs are different in clinical features and histopathologic findings, according to the presence or absence of combined endometriosis. We used seven histologic markers (PAX2, PAX8, ER, PR, WT1, CK20 and CDX2) commonly expressed in other types of ovarian tumors such as serous, mucinous or endometrioid.

## Results

### Comparison of clinicopathologic features according to combined endometriosis

Clinical features were compared between SMBT with and without endometriosis in Table 1. There were no statistical differences in clinical findings between two groups.

**Table 1:** Clinicopathologic features of patients with ovarian seromucinous borderline tumors with or without endometriosis

Characteristics	All (*N* = 69)	Without endometriosis (*N* = 29)	With endometriosis (*N* = 40)	*P* value
Age (yrs)	35.0 (30.0–43.0)	33.0 (30.0–44.5)	35.0 (30.2–41.5)	0.889
BMI (kg/m^2^)	20.7 (19.1–22.3)	20.9 (19.4–23.2)	20.5 (18.8–22.2)	0.508
Nulliparous	45 (65.2)	19 (65.5)	26 (65.0)	0.964
Menopause	9 (13.0)	4 (13.8)	5 (12.5)	1.000
Borderline op history	7 (10.1)	3 (10.3)	4 (10.0)	1.000
Endometriosis op history	3 (4.3)	0 (0)	3 (7.5)	0.258
ART history	4 (5.8)	2 (6.9)	2(5.0)	1.000
***Tumor and treatment***				
Serum CA125 (U/ml)	37.9 (17.6–62.9)	30.0 (17.1–56.5)	39.6 (17.3–63.6)	0.586
Normal	28 (44.4)	14 (53.8)	14 (37.8)	0.208
Elevated	35 (55.6) (*n* = 63)	12 (46.2) (*n* = 26)	23 (62.2) (*n* = 37)	
Tumor size (cm)	6.3 (4.5–8.3)	6.0 (4.1–9.5)	6.3 (4.6–8.2)	0.894
Surgical method				
Laparoscopy	65 (94.2)	26 (89.7)	39 (97.5)	0.302
Laparotomy	4 (5.8)	3 (10.3)	1 (2.5)	
Tumorectomy method				0.620
Cystectomy	19 (27.5)	9 (31.0)	10 (25.0)	
USO	38 (55.1)	14 (48.3)	24 (60.0)	
TH with BSO	12 (17.4)	6 (20.7)	6 (15.0)	
Staging operation				0.863
Not done	23 (33.3)	10 (34.5)	13 (32.5)	
Comprehensive	46 (66.7)	19 (65.5)	27 (67.5)	
Stage				0.167
1A	29 (42.0)	11 (37.9)	18 (45.0)	
1B	3 (4.3)	3 (10.3)	0 (0)	
1C	36 (52.2)	15 (51.7)	21 (52.5)	
2B	1 (1.4)	0 (0)	1 (2.5)	
Bilaterality of borderline tumor	5 (7.2)	3 (10.3)	2 (5.0)	0.643
Capsule involvement	5 (7.2)	4 (13.8)	1 (2.5)	0.154
Intraepithelial carcinoma	4 (5.8)	1 (3.4)	3 (7.5)	0.634
Microinvasion	9 (13.0)	4 (13.8)	5 (12.5)	1.000
Non- invasive implant	1 (1.4)	0 (0)	1 (2.5)	1.000
Peritoneal cytology	4 (10.8) (*n* = 37)	2 (14.3) (*n* = 14)	2 (8.7) (*n* = 23)	0.625
Pelvic adhesion	19 (27.5)	6 (20.7)	13 (32.5)	0.278
***Outcomes***				
Pregnancy outcome	9 (15.8) (*n* = 57)	2 (8.7) (*n* = 23)	7 (20.6) (*n* = 34)	0.288
Recurrence of borderline tumor	3 (4.3)	0 (0)	3 (7.5)	0.258
PFS, month	28.0 (12.5–60.0)	29.0 (11.5–60.0)	28.0 (12.5–61.0)	0.860
OS, month	29.0 (12.5–60.5)	29.0 (11.5–60.0)	28.0 (12.5–66.2)	0.831

Data were shown as median and interquartile ranges or numbers (percentages)

*EM* endometriosis, *BMI* body mass index, *ART* assisted reproductive technology, *USO* unilateral salpingo-oophrectomy, *TH* total hysterectomy, *BSO* bilateral salpingo-oophorectomy, *LND* lymph node dissection, *PFS* progression-free survival, *OS* overall survival

The median follow-up period was 29 months (range 12.5–60.5 months). During the follow-up period, a total nine of 57 (15.8%) women who performed fertility sparing surgery were pregnant and delivered the live baby. There was no significant difference in pregnancy outcome between SMBT with and without endometriosis (Table 1).

Three patients had SMBT recurrence, which was confirmed by surgery. All three patients with recurrence were seen in only ovarian SMBT with endometriosis, although there was no the statistical significance. The death in all patients was not shown during the follow-up period. There was no significant difference in progression-free survival (PFS) or overall survival (OS) between two groups (Table 1).

### Comparison of immunohistochemical features according to combined endometriosis

**Fig. 1:**
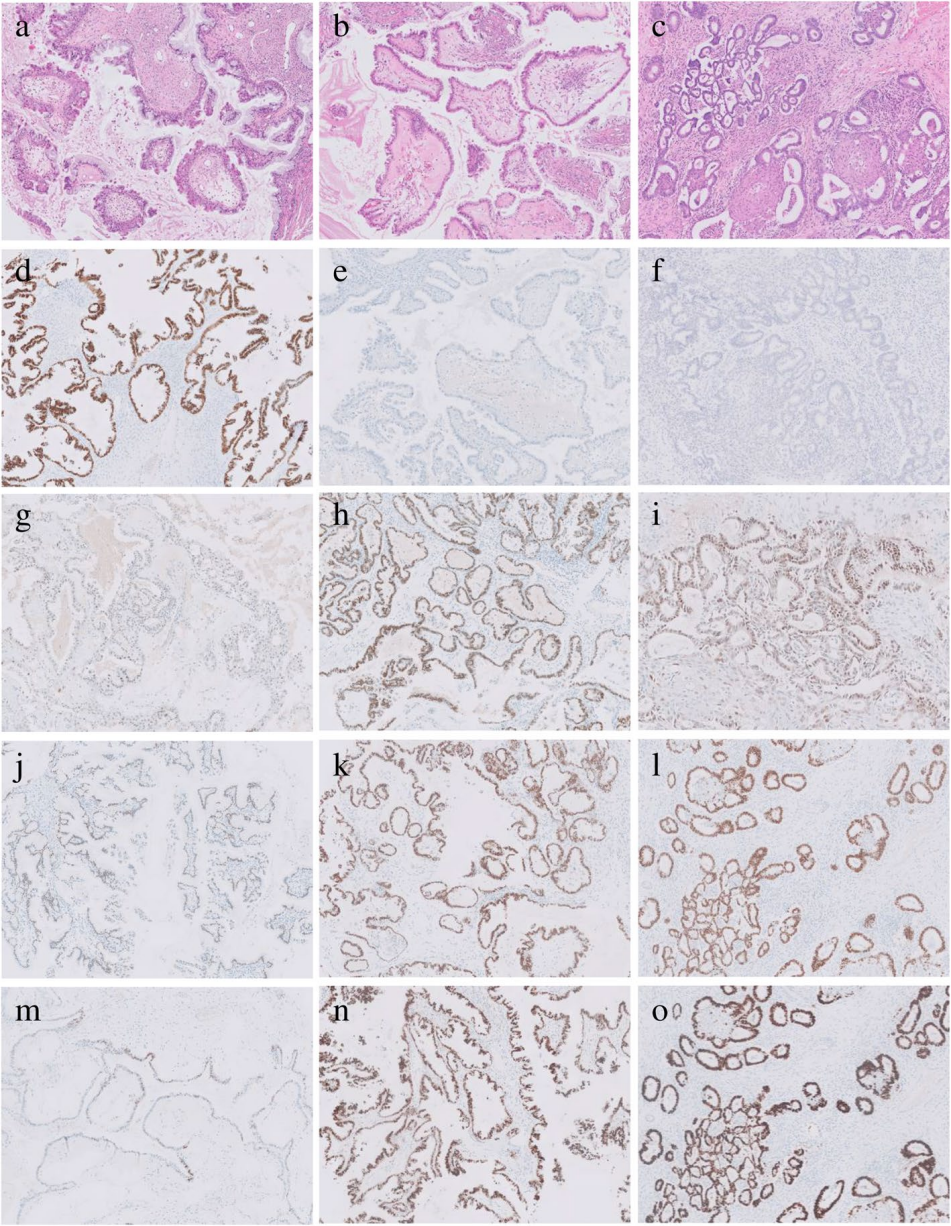
PAX2, PAX8 ER, and PR expressions in ovarian seromucinous borderline tumors and endometrioid borderline tumors. **a**-**c** Representative hematoxillin-eosin stained sections. **a** Seromucinous borderline tumor (SMBT) without endometriosis, **b** SMBT with endometriosis, and (**c**) endometrioid borderline tumor (EBT). **d** High PAX2 expression of SMBT without endometriosis. **e** Negative PAX2 expression of SMBT with endometriosis. **f** Negative PAX2 expression of EBT. **g** Low PAX8 expression of SMBT without endometriosis. **h** High PAX8 expression of SMBT with endometriosis. **i** High PAX8 expression of EBT. **j** High ER expression of SMBT without endometriosis. **k** High ER expression of SMBT with endometriosis. **l** High ER expression of EBT. **m** Low PR expression of SMBT without endometriosis. **n** High PR expression of SMBT with endometriosis. **o** High PR expression of EBT. (Original magnification, 100X in **a**-**o**)

Representative photomicrographs of PAX2, PAX8, ER, and PR expression in SMBT with and without endometriosis were shown in Fig. 1.

There was a difference in PAX2 and PAX8 expression between in ovarian SMBT with or without endometriosis. Eleven of 22 (50.0%) patients without endometriosis showed positive PAX2 expression, while eight of 36 (22.2%) patients with endometriosis showed positivity (*P* = 0.016). In contrast, all patients with endometriosis showed positive PAX8 expression, whereas only 14 of 22 (63.6%) patients without endometriosis showed positivity (*P* < 0.001) (Table 2).

**Table 2:** Immunohistochemical features in ovarian seromucinous borderline tumors with or without endometriosis

Positive Expression	Without endometriosis (*N* = 22)	With endometriosis (*N* = 36)	*P* value
PAX2	11 (50.0)	8 (22.2)	0.029
PAX8	14 (63.6)	36 (100.0)	< 0.001
ER	18 (81.8)	33 (91.7)	0.409
PR	9 (40.9)	26 (72.2)	0.018
WT1	8 (36.4)	8 (22.2)	0.242
CK20	0 (0)	1 (2.8)	1.000
CDX2	0 (0)	1 (2.8)	1.000

Data were shown as numbers (percentages)

PR positivity was more prominent in ovarian SMBT with endometriosis than without endometriosis (72.2% vs. 40.9%, *P* = 0.018), although there was no difference in positive ER expression. WT1, CK20 and CDX2 were scarcely expressed and were not different between ovarian SMBT with and without endometriosis (Table 2).

PAX2, PAX8, and PR expression in endometrioid borderline tumor (EBT) was also represented in Fig. 1. Their expressions in SMBT with endometriosis were similar to EBT unlike SMBT without endometriosis, although it was difficult to show the statistical significance due to small number of EBTs.

## Discussion

Since the seromucinous tumor has been adopted in 2014 WHO classification of ovarian tumor, the “seromucinous carcinoma” part was changed to the variant of endometrioid carcinoma in 2020 WHO classification [4]. However, the “seromucinous borderline tumor” part is still in the distinct category from “seromucinous carcinoma,” so two diseases may be considered as no more same lineage. SMBT has been one of the endometriosis-related ovarian tumors (EROT) similar to ovarian clear cell carcinoma and endometrioid carcinoma, while clear cell borderline tumor or endometrioid borderline tumor are very rare and are difficult to discuss whether both are representative tumor of EROT. In this study of total 162 patients who underwent surgical excision of the ovarian borderline tumor between January 2009 and January 2020 at a single institution, only two patients were diagnosed as EBT, and there was no clear cell borderline tumor. Meanwhile, SMBT were diagnosed in 69 patients (42% of all borderline tumors), and 58% of SMBT were found with combined endometriosis.

This study showed that there was no difference in clinical features of ovarian SMBT according to the combined endometriosis, which may be postulated to be due to the fact that they are all borderline tumors. The presence or absence of endometriosis cannot be defining factors to have an impact on clinical outcomes. But it should be noted that, although there was no statistical significance, all three recurred cases were SMBT with endometriosis. Even serum CA125, endometriosis operation history, or history of assisted reproductive technology (ART), which are known as typical endometriosis features, were not different. Fertility sparing surgery was performed in 82% of patients with ovarian SMBT including even cystectomy in 27% of patients, because most women were still young, whose median age was 35 years. The median age of SMBT in this study was slightly younger than in other literatures, which showed more late reproductive ages as 45–47 years [5, 6] or even 63.2 years [7]. However, these studies had relatively smaller number of cases than the present study, and large cohort of ovarian borderline tumor reported that the mean age was 36–38 years [8], similar to this study.

Pregnancy outcomes with live birth between two groups were not significantly different. This study precisely lacked the intention to conceive or the rate of abortion, but the real live birth outcomes was suggested in fertility sparing surgery group regardless of effort for pregnancy. Approximately 15% women with fertility sparing surgery normally delivered, and 20% women with combined endometriosis had live birth, which was slightly higher than the no endometriosis group, although there was no statistical significance. In all types of borderline tumor, it was reported that about 42% of live birth rate after fertility sparing surgery and 90.7% were free of recurrence [9].

Recently, new Müllerian markers (PAX2 and PAX8) are reported as useful markers to differentiate Müllerian mucinous tumors from non- Müllerian tumors [10–12]. PAX2 and PAX8 belong to the pair box gene family consisting of nine members (PAX1 to PAX9), each of which encodes a transcription factor [11]. These are expressed during fetal development and known to control the development of organs deriving from the Müllerian duct such as the fallopian tube, endometrium, and endocervix, but not the development of the ovary [13, 14]. In our study, a few cases of SMBT with combined endometriosis showed positive expression of PAX2 and most cases showed positive expression of PAX8 in contrast to SMBTs without endometriosis. In ovarian tumors, PAX2 is detected in clear cell and mucinous tumors, but absent in most endometrioid tumors [15–17], while PAX8 shows high expression in clear cell and endometrioid tumors and reduced expression in mucinous tumors [18–20]. In the same seromucinous tumor category, there was no uniform pattern in PAX2 and PAX8 expression. However, when the seromucinous tumor was divided according to the presence or absence of endometriosis, a unique expression pattern was shown.

SMBT with endometriosis in this study showed similar expression patterns of PAX2 and PAX8 with those in endometrioid tumors, in contrast to SMBT without endometriosis. The exact mechanism explaining how endometriosis is related to the development or progression of ovarian SMBT is not clarified yet, however, it has become clear that Müllerian markers such as PAX2 and PAX8 show distinctively different expression pattern between SMBTs with and without endometriosis. In this study, PAX2 and PAX8 patterns in two patients with EBT was seen. PAX2 and PAX8 expressions in SMBT with endometriosis were closer to the expression patterns in EBT, implicating that SMBT with endometriosis might also be associated with endometrioid tumors like ex- “seromucinous carcinoma” and may be different from SMBT without endometriosis in a specific molecular pattern.

Highly positive ER expression was found in both SMBT with and without endometriosis. In contrast, PR expression was higher in SMBT with endometriosis than without endometriosis. Other literature reported that both ER and PR positivity were shown in SMBT, while negative for ER and PR in mucinous borderline tumor [21]. In a recent study, hormone receptor expression, especially PR expression, is most common in endometrioid tumors but almost absent in clear call and mucinous carcinomas [22, 23]. PR patterns of ovarian SMBT with endometriosis in this study is also close to EBTs, suggesting ovarian SMBT with endometriosis may be a part of endometrioid tumors unlike SMBT without endometriosis.

The Wilms tumor 1 (WT1) gene was discovered as a tumor suppressor gene in Wilms tumor [24]. In gynecology, WT1 expression has been used for diagnostic purposes of serous type tumors [25]. Both CK20 and CDX2 were expressed in mucinous tumors, and commonly used for distinguish metastatic ovarian mucinous tumors from primary ovarian mucinous tumors [26]. Ovarian SMBT in this study showed no or low expression of all serous and mucinous markers, which means that SMBTs are not close to serous or mucinous characteristics.

A limitation of this study was the narrow cohort, which was only confined to a single institution. In addition, no scoring system was used for the interpretation of immunohistochemical staining, which was not delicate. The other of limitation is that this is not the direct comparison study between ovarian SMBT and EBT, due to the rarity of EBT. However, this study discussed the findings of larger numbers of ovarian SMBTs than other literatures, and it can conclude the more objective features of these tumors.

In conclusion, only a few cases of ovarian SMBT with endometriosis showed expression of PAX2 and conversely, most of the cases of ovarian SMBTs with endometriosis showed expression of PAX8 in contrast to SMBTs without endometriosis. PR positivity was also more prominent in ovarian SMBT with endometriosis than without endometriosis. Ovarian SMBT with endometriosis showed the similar immunohistochemical patterns to endometrioid tumors unlike SMBT without endometriosis even though SMBT with endometriosis and without endometriosis have same clinical patterns. It suggests that just as seromucinous carcinoma has been removed from the seromucinous category and newly defined as a subtype of endometrioid carcinoma, SMBT with endometriosis may also be a borderline counterpart of seromucinous carcinoma, and may have to be removed from the seromucinous category and put under the endometrioid borderline category as a subtype. To validate this hypothesis, further comparison studies between seromucinous borderline tumor and endometrioid tumor are needed with gene profiling studies to prove their relationship.

## Methods

### Tumors and patients

This retrospective study was approved by the institutional review board (IRB) of CHA Gangnam Medical Center (No. GCI 2020–04-010–001) and conducted in accordance with the principles of the Declaration of Helsinki. The requirement for informed consent was waived by the IRB due to its retrospective design.

Patients who underwent surgical excision of ovarian borderline tumors between January 2009 and January 2020 at CHA Gangnam Medical Center were identified and selected for review. A total of 162 patients underwent surgical excision of the ovarian borderline tumor in this period. In accordance with the new 2014 WHO classification of ovarian tumors, hematoxylin- and eosin-stained slides of all selected cases were reviewed by two pathologists, and all patients were reclassified according to the criteria. Two of 26 patients with ovarian serous borderline tumor were confirmed as SMBT and 17 of 84 patients with ovarian mucinous borderline tumor were diagnosed as SMBT. The two EBTs were confirmed to be the same type. Finally, 69 of 162 patients with borderline tumors were diagnosed as having ovarian SMBT and was included in the analysis, 40 of whom (58.0%) with ovarian SMBT showed combined endometriosis and 29 did not.

Clinical information of patients was obtained from medical records. Clinical information on age, body mass index, parity, pre-/post-menopause, history of ovarian borderline tumor surgery, history of ovarian endometriosis surgery, history of ART, serum CA125 levels (normal range: 0–35 U/mL), and tumor size by ultrasonography were evaluated. Surgical method was laparoscopy or laparotomy. Tumorectomy method was cystectomy, unilateral salpingo-oophorectomy or total hysterectomy with bilateral salpingo-oophorectomy. Staging operation was divided as not done or comprehensive surgical staging (including omentectomy, appendectomy, peritoneal biopsy, peritoneal cytology or lymph node dissection). Staging was re-evaluated by 2014 International Federation of Gynecology and Obstetrics system. The presence of tumor in contralateral ovary was also evaluated, and the tumor type was divided into borderline, benign or endometrioma. The presence of capsule involvement, histologic presence of intraepithelial carcinoma or microinvasion, the presence of peritoneal implant, positivity of peritoneal cytology, and pelvic adhesion of operative field were evaluated.

After surgery, the presence of pregnancy and the recurrence of borderline tumor or carcinoma were evaluated. PFS was defined as the period from the day of first surgery to the day of recurrence. OS was defined as the period from the day of first surgery to that of last contact alive.

### Tissue microarray construction

Tissue microarray (TMA) was constructed in 58 of 69 (84.1%) patients with ovarian SMBT due to the issues with the quality of paraffin block, and also in two patients with EBT. Representative areas were selected on a hematoxylin- and eosin-stained slide of each case, and corresponding areas on the matching paraffin block were marked. Using a manual device, three 2 mm-sized cores from representative areas of each case were punched from the donor block and transferred to the 6 × 10 recipient block.

### Immunohistochemistry

Only 3 μm thick tissue sections of each TMA block were cut, deparaffinized and rehydrated in xylene, and graded alcohol. Ventana Discovery XT automated stainer (Ventana Medical Systems, Tucson, AZ, USA) was used for immunohistochemical staining. Primary antibodies used were as follows: PAX2 (Abcam, USA), PAX8 (Roche, Switzerland), ER (Novocastra, England), PR (Novocastra, England), WT-1 (Novocastra, England), CK20 (Dako, Denmark) and CDX2 (Cell marque, USA).

### Interpretation of immunohistochemical staining

Epididymis tissue was used as a positive control for PAX2 and kidney tubules as a positive control for PAX8. Invasive ductal carcinoma of the breast with Allred score 8 for ER and PR was used as positive controls for ER and PR staining. Mesothelioma tissue was used as a positive control for WT-1, and colon cancer tissue was used as a positive control for both CK20 and CDX2. Each IHC staining intensity was graded at first as follows: complete negative, 0; weak staining, 1 + ; moderate, 2 + ; and strong, 3 + , as in invasive ductal carcinoma of the breast. However, most of the cases that stained positive were grade 2 or 3 and thus grading was of little significant value. The cases were instead subgrouped into positive or negative staining groups.

### Statistical analysis

The numerical variables were presented as the median and interquartile range after checking the Kolmogorov–Smirnov normality test. The differences of the groups were evaluated using the Mann–Whitney test for continuous variables. For categorical variables, the χ^2^ test was used. *P* value < 0.05 was considered statistically significant. The statistical analysis was performed using SPSS ver. 26.0 (IBM Corp., Armonk, NY, USA).

## Data Availability

The dataset used and/or analyzed during the current study are available from the corresponding authors on reasonable request.

## Abbreviations

SMBT: Seromucinous borderline tumor
EBT: Endometrioid borderline tumor
PFS: Progression-free survival
OS: Overall survival
EORT: Endometriosis-related ovarian tumors
ART: Assisted reproductive technology
IRB: Institutional review board
TMA: Tissue microarray
